# Correction to “Serious mental illness and cancer: A call to action for equitable care”

**DOI:** 10.3322/caac.70101

**Published:** 2026-07-30

McFarland D, Bergerot C, Bidstrup P, et al. Serious mental illness and cancer: a call to action for equitable care. *CA Cancer J Clin*. 2026;e70095. doi:10.3322/caac.70095

An author’s first name was misspelled in the original publication. The sixth author’s name should read Shahrzad Zamani.

The authors apologize for this error.

